# Transmembrane protein sorting driven by membrane curvature

**DOI:** 10.1038/ncomms9728

**Published:** 2015-11-02

**Authors:** H. Strahl, S. Ronneau, B. Solana González, D. Klutsch, C. Schaffner-Barbero, L. W. Hamoen

**Affiliations:** 1 Centre for Bacterial Cell Biology, Institute for Cell and Molecular Biosciences, Newcastle University, Richardson Road, Newcastle upon Tyne NE2 4AX, UK; 2 Swammerdam Institute for Life Sciences (SILS), University of Amsterdam, 1098 XH Amsterdam, The Netherlands

## Abstract

The intricate structure of prokaryotic and eukaryotic cells depends on the ability to target proteins to specific cellular locations. In most cases, we have a poor understanding of the underlying mechanisms. A typical example is the assembly of bacterial chemoreceptors at cell poles. Here we show that the classical chemoreceptor TlpA of *Bacillus subtilis* does not localize according to the consensus stochastic nucleation mechanism but accumulates at strongly curved membrane areas generated during cell division. This preference was confirmed by accumulation at non-septal curved membranes. Localization appears to be an intrinsic property of the protein complex and does not rely on chemoreceptor clustering, as was previously shown for *Escherichia coli*. By constructing specific amino-acid substitutions, we demonstrate that the preference for strongly curved membranes arises from the curved shape of chemoreceptor trimer of dimers. These findings demonstrate that the intrinsic shape of transmembrane proteins can determine their cellular localization.

## Abstract (web summary)

The accumulation of chemoreceptor proteins at bacterial poles is thought to depend on their clustering into arrays. Strahl *et al.* show that in *Bacillus subtilis*, the chemoreceptor TlpA uses high membrane curvature as a spatial cue for polar localization, through the intrinsic curvature sensitivity of the receptor complex.

## Graphical abstract

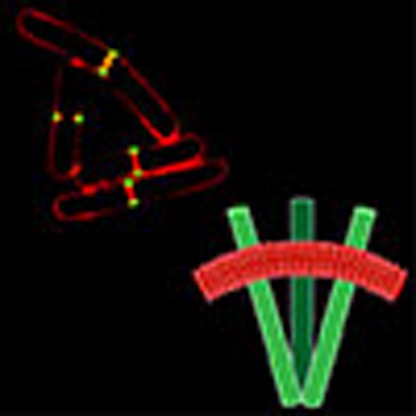

Cells rely on a strict cellular organization for their growth and function, and many biochemical processes are confined to specific areas in the cell^1^,^2^. In rod-shaped bacteria, the cell pole is such an area to which a large number of proteins are recruited^2^,^3^,^4^,^5^. Despite its importance, we still do not have a good understanding by which mechanisms proteins are sequestered to the bacterial cell poles. A classic example is the polar localization pattern of the chemotactic sensory complexes^6^,^7^.

Bacteria sense their surrounding by transmembrane or cytoplasmic chemoreceptor proteins that form dimers, which assemble into trimers. Together with specific kinases, phosphatases and receptor-modulating proteins, they form large stable sensory clusters. The assembly of individual sensory proteins into large clusters provides high sensitivity, and allows the cell to integrate various environmental signals into a unified output^8^,^9^. These intricate signal transduction complexes regulate flagellar rotation, thereby controlling the swimming direction of bacteria^10^,^11^. In their seminal paper >20 years ago, Maddock and Shapiro showed that bacterial chemoreceptor clusters are specifically localized at cell poles in *Escherichia coli*^7^. This discovery led to the realization that bacterial cells are far more organized than was previously anticipated. This polar localization pattern has later been confirmed in many other bacterial species. Despite the fact that the conserved chemosensory signalling complexes have been extensively studied, it is still not fully clear how they assemble at the bacterial cell poles.

Two models have been postulated as potential mechanisms for polar targeting. According to the ‘stochastic nucleation’ model, the new chemoreceptor clusters are formed primarily at midcell. The reason for this is that with increasing distances from pre-existing polar clusters, the newly synthesized chemoreceptor proteins are more likely to nucleate new clusters rather than merge with polar clusters. Thus, the new chemoreceptor clusters are present at the proper location even before cell division is initiated (at midcell) and the new cell poles are formed^12^,^13^,^14^,^15^. In the alternative model, large chemoreceptor clusters accumulate at the curved cell poles due to the slightly curved shape of the chemoreceptor trimer of dimers as this reduces ‘curvature mismatch’^16^. Since individual chemoreceptors are more than an order of magnitude smaller than the cell pole, this mechanism requires that the chemoreceptor proteins cluster into large chemosensory arrays. The model also predicts that the formation of clusters along the lateral cell wall is suppressed, and therefore the model is incompatible with the stochastic nucleation model^16^.

Thus far, the *in vivo* data support the stochastic nucleation model^12^,^13^,^14^. To examine whether this mechanism is also active in other rod-shaped bacteria such as the Gram-positive model organism *Bacillus subtilis*, we followed the localization of a typical chemoreceptor protein in this organism. Surprisingly, no regular periodic clustering and no direct recruitment to the cell pole were observed. In contrast, the polar localization pattern turned out to be a consequence of recruitment to cell division sites. After cell division is completed, chemoreceptors remain located at the newly formed cell poles. On the basis of extensive microscopic and mutational analyses, we put forward a new model for polar chemoreceptor localization based on the highly curved septal membrane as a spatial cue. The similarities and differences with existing localization models are discussed.

## Results

### Cellular localization of the *B. subtilis* chemoreceptor TlpA

**Figure 1:**
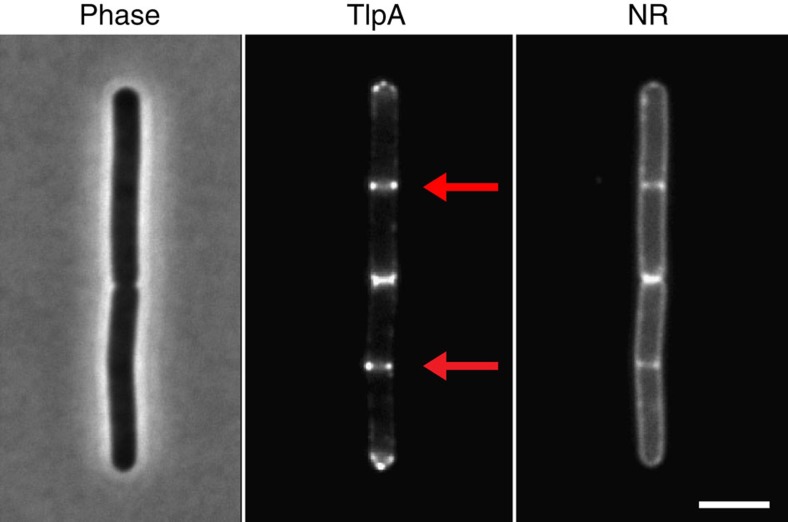
TlpA localizes at cell poles and cell division sites. Phase-contrast image of *B. subtilis* cells (left panel) expressing TlpA-GFP (middle panel), and stained with the fluorescent membrane probe Nile red (right panel). The active cell division sites are highlighted with an arrow. Strain used: *B. subtilis* HS48 (*Pxyl-tlpA-gfp*). Scale bar, 3 μm.

To analyse chemoreceptor clustering in *B. subtilis*, we followed the localization of a typical chemoreceptor protein, TlpA, by constructing a C-terminal green fluorescent protein (GFP) fusion. Dimerization of GFP can cause artificial polar localization of proteins^17^, therefore a monomeric variant of GFP was used. As shown in Fig. 1 and Supplementary Fig. 1a, the TlpA-GFP fusion accumulated at midcell and cell poles, as has been reported before^18^. Different chemoreceptors form mixed clusters. The polar localization of TlpA-GFP could therefore be caused by incorporation into existing polar chemoreceptor clusters. However, a *B. subtilis* strain lacking all 10 native chemoreceptors showed exactly the same localization pattern (Supplementary Fig. 1b), indicating that the TlpA-GFP fusion does not require other chemoreceptors for recruitment.

### Cell division-dependent clustering of TlpA

**Figure 2:**
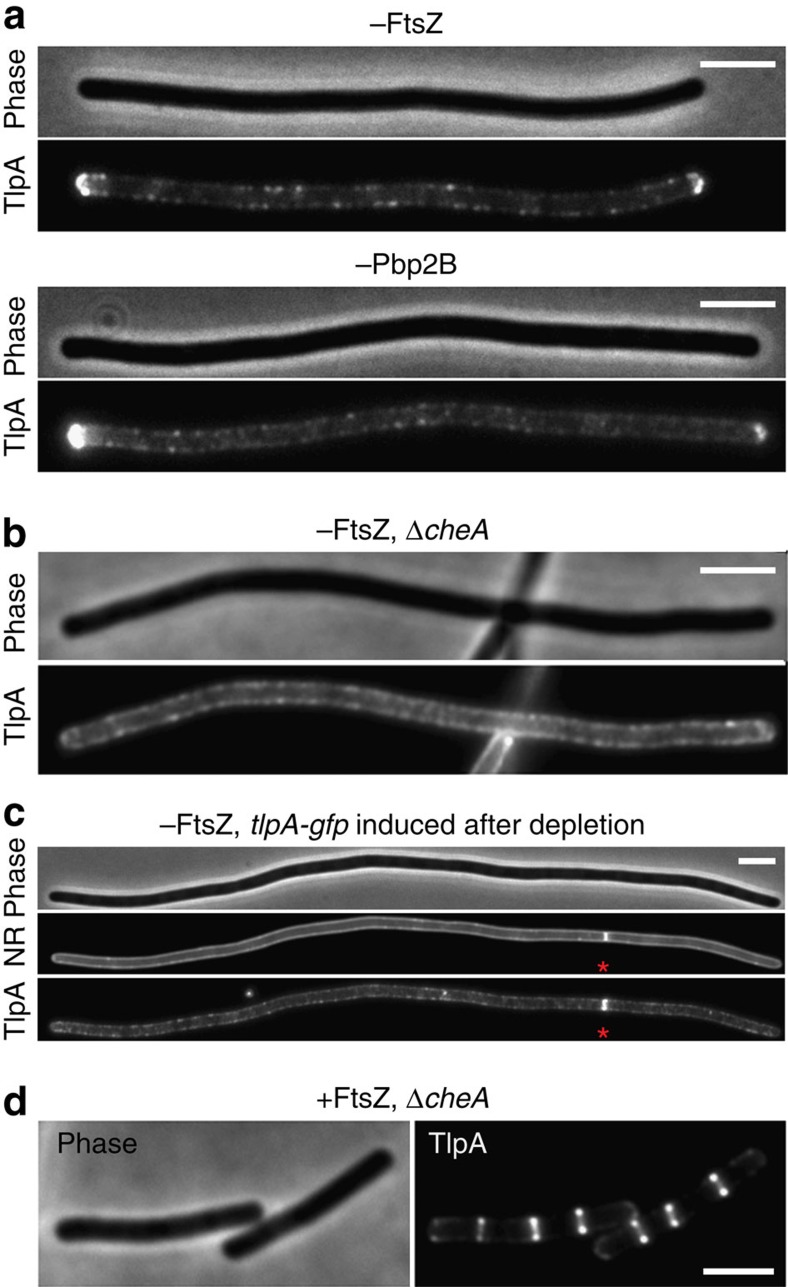
Localization of TlpA is cell division dependent. (**a**) Midcell localization of TlpA-GFP is abolished in cells depleted for FtsZ (upper panel) and Pbp2B (lower panel). (**b**) Polar accumulation of TlpA-GFP is absent in cells depleted for FtsZ when *cheA* is deleted. (**c**) No recruitment to cell poles is observed when TlpA-GFP is induced after FtsZ depletion. However, a clear recruitment is observed to a single cell division site that is still present (indicated with an asterisk). (**d**) TlpA-GFP still localizes at cell division sites in a *cheA* deletion mutant. Strains used: (**a**) *B. subtilis* HS50 (Δ*mcp Pxyl-tlpA-gfp Pspac-ftsZ*), *B. subtilis* HS51 (Δ*mcp Pxyl-tlpA-gfp Pspac-pbpB*), (b/d) *B. subtilis* HS52 (Δ*cheA Pxyl-tlpA-gfp Pspac-ftsZ*) and (**c**) *B. subtilis* HS50 (Δ*mcp Pxyl-tlpA-gfp Pspac-ftsZ*). Scale bar, 3 μm.

For *E. coli*, it was shown that the methyltransferase CheR and the response regulator CheY, which in these studies were used as proxy for chemoreceptor clusters, accumulate into large regularly spaced clusters along the cell axis even when cell division is inhibited (stochastic nucleation model)^14^. Subsequent studies indicated that these periodically spaced clusters can arise spontaneously and do not require tethering to a pre-existing structure^13^,^15^. To test whether TlpA also shows a periodic localization pattern when cell division is inhibited, *B. subtilis* cells were depleted for the essential cell division protein FtsZ. As shown in Fig. 2a and Supplementary Fig. 2a, many small fluorescent protein clusters are scattered along the cell membrane but no large regularly spaced clusters were found. Only at the cell poles does TlpA-GFP form a large cluster. We repeated the depletion experiment with Pbp2B, a penicillin-binding protein which is essential for synthesis of the septal cell wall. In the absence of this protein, the septum constriction is blocked although the cell division machinery is correctly assembled^19^. Depletion of Pbp2B resulted in the same TlpA-GFP localization pattern as with FtsZ depletion (Fig. 2a; Supplementary Fig. 2b).

### Cluster distribution

Clustering of chemoreceptors is mediated by binding of the histidine kinase CheA- and the CheW-coupling protein^20^,^21^. To confirm that the observed TlpA foci are clusters, we carried out a co-localization analysis. Indeed, CheA fluorescently labelled with mCherry showed a clear co-localization with TlpA-GFP foci (Supplementary Figs 3 and 4). To further confirm this, the FtsZ-depletion experiment was repeated in *cheA* and *cheW* deletion strains. As shown in Fig. 2b, Supplementary Figs 2d,e and 5, the absence of CheA or CheW abolishes distinct polar TlpA clusters and results in a considerably smoother fluorescence signal along the lateral cell wall.

To have a more detailed understanding of the chemoreceptor cluster distribution, we measured fluorescence intensities along the length of FtsZ-depleted cells. A visual inspection did not reveal an obvious regularity in the peak pattern (Supplementary Figs 5 and 6). A more quantitative assessment using Fast Fourier analysis gave also no indication for a periodic spacing between chemoreceptor clusters (Supplementary Fig. 7). A quantification of clusters in several FtsZ-depleted cells resulted in an average number of 4.5±0.5 clusters per micrometre. This amounts to ∼22 clusters per cell, when assuming an average cell length for *B. subtilis* of 5 μm (Supplementary Fig. 8). Because of this relative large number and the random distribution of clusters, a stochastic nucleation mechanism, which predicts two to four clusters per cell^12^,^13^,^15^, appears to be inadequate to explain polar localization of TlpA.

### Polar targeting

As shown in Fig. 2b, when CheA is absent, the strong polar TlpA-GFP signal disappears as well (Supplementary Fig. 2d,e). In fact, it was already shown for *E. coli* that CheA is required for the clustering of chemoreceptors at cell poles^22^. This finding led to an alternative model for polar localization of chemoreceptors; one that is based on the fact that chemoreceptors themselves are slightly curved^16^. Bacterial chemoreceptor molecules form dimers that assemble into a stable trimeric complex. The individual dimers do not run parallel but fan out from the cytoplasmic interaction domain and form a tripod-like structure, as a consequence of which the trimer of dimers is slightly curved^9^,^16^,^20^,^23^. At the cell poles, the cytoplasmic membrane is curved in two dimensions, whereas the lateral cytoplasmic membrane is curved in only one dimension (Supplementary Fig. 9). This difference in curvature is very small since the radius of the cell and the radius of the cell pole are comparable, and it is unlikely that individual chemoreceptor trimer of dimers can distinguish this curvature difference. However, mathematical modelling has shown that, in theory, chemoreceptors could sense this slight difference in curvature when they assemble into large clusters with the help of CheA and CheW^16^. The absence of polar TlpA-GFP clusters in *cheA* and *cheW* deletion mutants appears to confirm this theory. However, if this localization mechanism is correct, then expression of TlpA-GFP after blockage of cell division should still result in polar enrichment. To test this, FtsZ was first depleted resulting in long cells. When this stage was reached, TlpA-GFP was induced. As shown in Fig. 2c and Supplementary Fig. 2c, the fluorescent clusters are now spread all over the cell membrane and there is no clear enrichment at cell poles anymore. On the basis of these data, we must assume that, at least in *B. subtilis*, chemoreceptor clusters do not have a specific affinity for cell poles. Apparently, neither stochastic nucleation nor curvature dependency of chemoreceptor clusters can explain the polar accumulation of TlpA.

### TlpA localizes at the base of the septum

**Figure 3:**
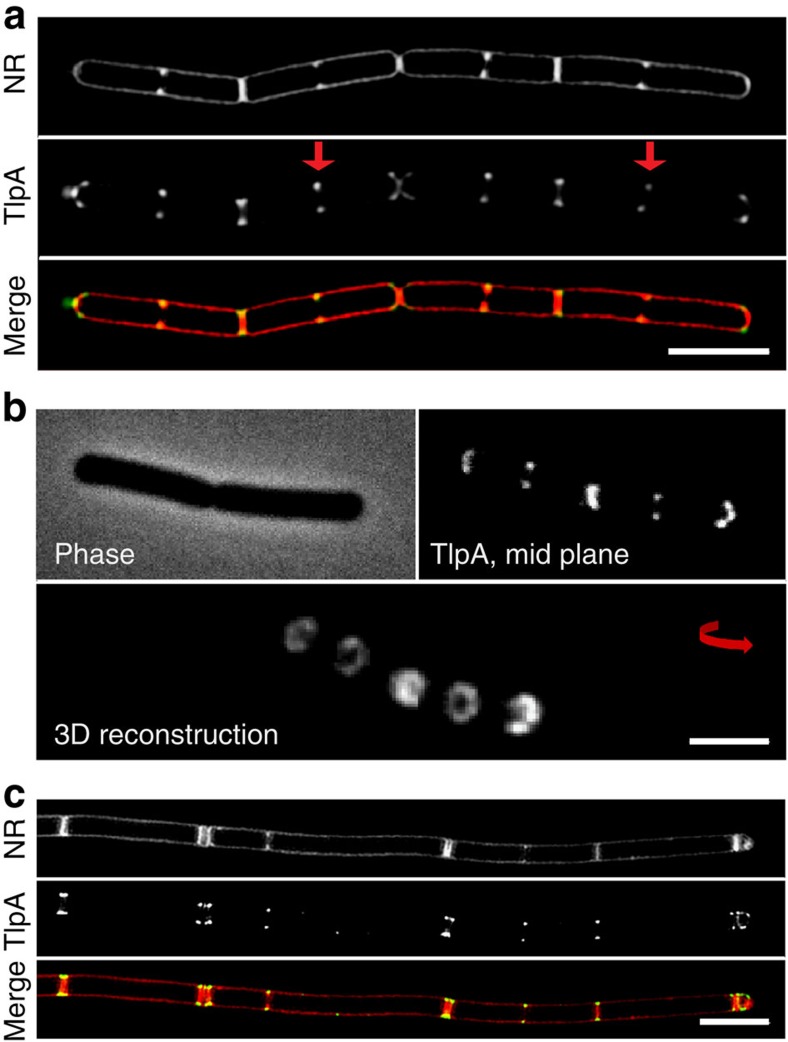
TlpA localizes to strongly curved septal membranes. (**a**) Structured illumination microscopy (3D SIM) images of *B. subtilis* cells stained with Nile red (upper panel) and expressing TlpA-GFP (middle panel). Merged image shown in lower panel. (**b**) Phase-contrast image (upper left panel), confocal fluorescence image (upper right panel) and 3D reconstruction of optical sectioning of cells expressing TlpA-GFP (lower panel). (**c**) Localization of TlpA-GFP in the absence of DivIVA. 3D SIM images of Nile red membrane stained *divIVA* deletion mutant (upper panel) expressing TlpA-GFP (middle panel), and merged image (lower panel) are depicted. In the absence of DivIVA, *B. subtilis* cells frequently undergo multiple adjacent cell division events^60^,^61^. Strains used: (**a**,**b**) *B. subtilis* HS49 (Δ*mcp Pxyl-tlpA-gfp*) and (**c**) *B. subtilis* HS52 (Δ*mcp Pxyl-tlpA-gfp* Δ*divIVA*). Scale bar, 3 μm.

To look into more detail at the TlpA-GFP localization, we employed super-resolution structured illumination (SIM) microscopy. As shown in Fig. 3a, the GFP signal appears at midcell when the membrane starts to invaginate. Interestingly, even when the septum divides the cell completely, the fluorescent signal remains bilaterally at the base of the division septum, and does not cover the septal membrane (Supplementary Fig. 10). The resulting ring-like localization pattern of TlpA-GFP is also clearly seen in three-dimensonal (3D)-reconstructed confocal Z-sections (Fig. 3b).

The transition from the lateral wall to the septum, where TlpA accumulates, is characterized by a strongly curved cell membrane. The cell division and cell growth regulator DivIVA, a conserved peripheral membrane protein in Gram-positive bacteria, is able to use this topological characteristic for its localization, and a DivIVA-GFP fusion shows a localization pattern reminiscent to that of TlpA-GFP^24^,^25^. DivIVA recruits a wide variety of proteins, however, Fig. 3c and Supplementary Fig. 11a indicate that the localization of TlpA-GFP does not change in a *divIVA* deletion mutant. Certain lipid species such as cardiolipin (CL) and phosphatidyl-ethanolamine (PE) are also enriched at cell division sites^26^. A polar recruitment of chemoreceptors based on preferential interactions with these lipid species has been postulated before^27^, but this mechanism was recently ruled out for *E. coli*^28^. Indeed, *B. subtilis* deletion mutants that lack these lipid species also show a normal TlpA localization (Supplementary Fig. 11b).

### TlpA is recruited to curved membranes

**Figure 4:**
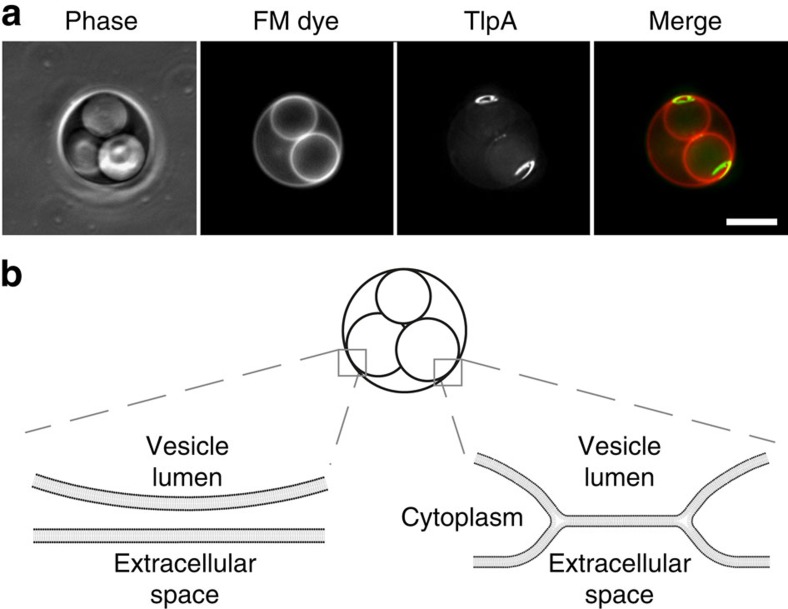
TlpA is recruited to non-septal curved membranes. (**a**) The differential staining of internal vacuole-like structures with membrane dye FM 4–64 in *B. subtilis* L-forms indicates the presence of two distinct types of vacuoles within the cell^32^. The membrane dye FM 4–64 is not able to cross biological membranes and therefore does not stain internal membrane structures, unless they remain connected to the cytoplasmic membrane^62^,^63^. The connection with the cytoplasmic membrane (membrane hemifusion) generates membrane areas with a distinct curvature. TlpA-GFP is strongly recruited to these non-septal areas of high membrane curvature. Maximal intensity projection of a deconvolved optical sectioning is shown. Strain used: *B. subtilis* HS55 (*Pxyl-tlpA-gfp* L-form). Scale bar, 5 μm. (**b**) Schematic depiction of the interfaces between the vacuolar and cytoplasmic membranes. In the left, the membranes are fully separated resulting in a lack of FM 4–64 staining. In the right, the membranes remain attached via membrane hemifusion, allowing lateral diffusion of FM 4–64.

Septal localization of TlpA depends neither on chemoreceptor clustering (CheA and CheW) or the scaffold protein DivIVA nor on lipids such as CL or PE, and it might therefore be an intrinsic property of the protein. Possibly, the inherent curvature of chemoreceptor trimer of dimers is still a valid explanation for the accretion of TlpA at the strongly curved membrane areas that are formed by the cell division process. This despite the fact that we have shown that curved chemoreceptor clusters are not able to distinguish polar curvature from the curvature of the lateral membrane (Fig. 2c; Supplementary Fig. 2c). On the basis of structural data, it was calculated that the curvature of chemoreceptor trimers, when measured from membrane mid-plane to cytoplasmic tip, amounts to a radius of ∼37 nm (ref. 16). This approaches the radius of the highly curved membrane area at the base of cell division septa that have been estimated at ∼47 nm (ref. 24). Importantly, this area is almost ten times more curved compared with the lateral cell membrane (cell diameter∼800 nm). Several theoretical studies have suggested that membrane proteins that deform the lipid bilayer are likely to segregate into regions of their preferred curvature, to relax stresses in the membrane^29^,^30^. Thus, it might be that chemoreceptor trimer of dimers prefer the strong concave membrane areas at the base of division septa. Support for this model was obtained when we expressed TlpA-GFP in *B. subtilis* L-form cells^31^. The induction of this cell-wall-less state results in the formation of large intracellular vesicles connected to the cytoplasmic membrane^32^. As shown in Fig. 4, a strong enrichment of TlpA was observed at highly curved vesicle-to-cytoplasmic membrane interfaces in the L-form cell.

### Recruitment is driven by the shape of chemoreceptor trimers

**Figure 5:**
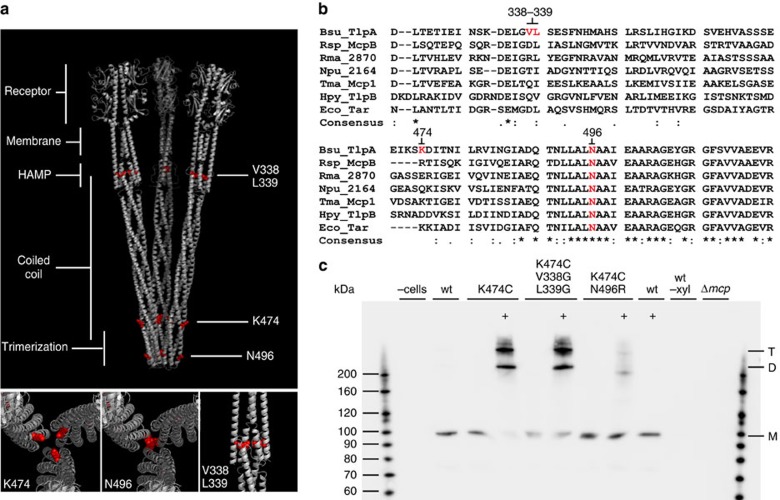
Complex formation analysis of TlpA mutants by *in vivo* crosslinking. (**a**) Structural model of TlpA trimer of dimers with the position of the amino-acid substitutions highlighted in red, and close-up images of the same substitutions shown below. (**b**) ClustalW2 alignment of selected chemoreceptors representing examples from different bacterial phyla and classes. Bsu, *B. subtilis* (Firmicutes); Eco, *E. coli* (φ-Proteobacteria); Npu, *Nostoc punctiforme* (Cyanobacteria); Rsp, *Rhodobacter sphaeroides* (α-Proteobacteria); Rma, *Rhodothermus marinus* (Bacterioidetes); Tma, *Thermotoga maritima* (Thermatogae); Hpy, *Helicobacter pylori* (ε-Proteobacteria). The positions of the introduced amino-acid substitutions are highlighted in red. Note that the introduction of two glycine substitutions at positions 338 and 339 creates a stretch of three glycines. (**c**) Western blot of TlpA-GFP complexes carrying the N_496_R, and V_338_G L_339_G exchanges after *in vivo* crosslinking with TMEA. A cysteine residue was introduced at position 474, which allows tris(2-maleimidoethyl) amine (TMAE) crosslinking of neighbouring TlpA dimers if those are assembled into a trimer of dimers. Crosslinked samples are highlighted with ‘+’. As a control, Δ*mcp* cells encoding wild-type TlpA-GFP with induction (wt), without induction (wt -xyl) and the parental strain (Δ*mcp*) are shown. Monomeric TlpA-GFP (99 kDa), and crosslinked dimer and trimer bands are highlighted with M, D and T, respectively. Strains used: *B. subtilis* 168 (wild type), *B. subtilis* OI3545 (Δ*mcp*)*, B. subtilis* HS49 (Δ*mcp pxyl-tlpA-gfp*), *B. subtilis* HS58 (Δ*mcp pxyl-tlpA(K*_*474*_*C)-gfp*), *B. subtilis* HS59 (Δ*mcp pxyl-tlpA(K*_*474*_*C, N*_*496*_*R)-gfp*) and *B. subtilis* HS60 (Δ*mcp pxyl-tlpA(K*_*474*_*C, V*_*338*_*G, L*_*339*_*G)-gfp*).

**Figure 6:**
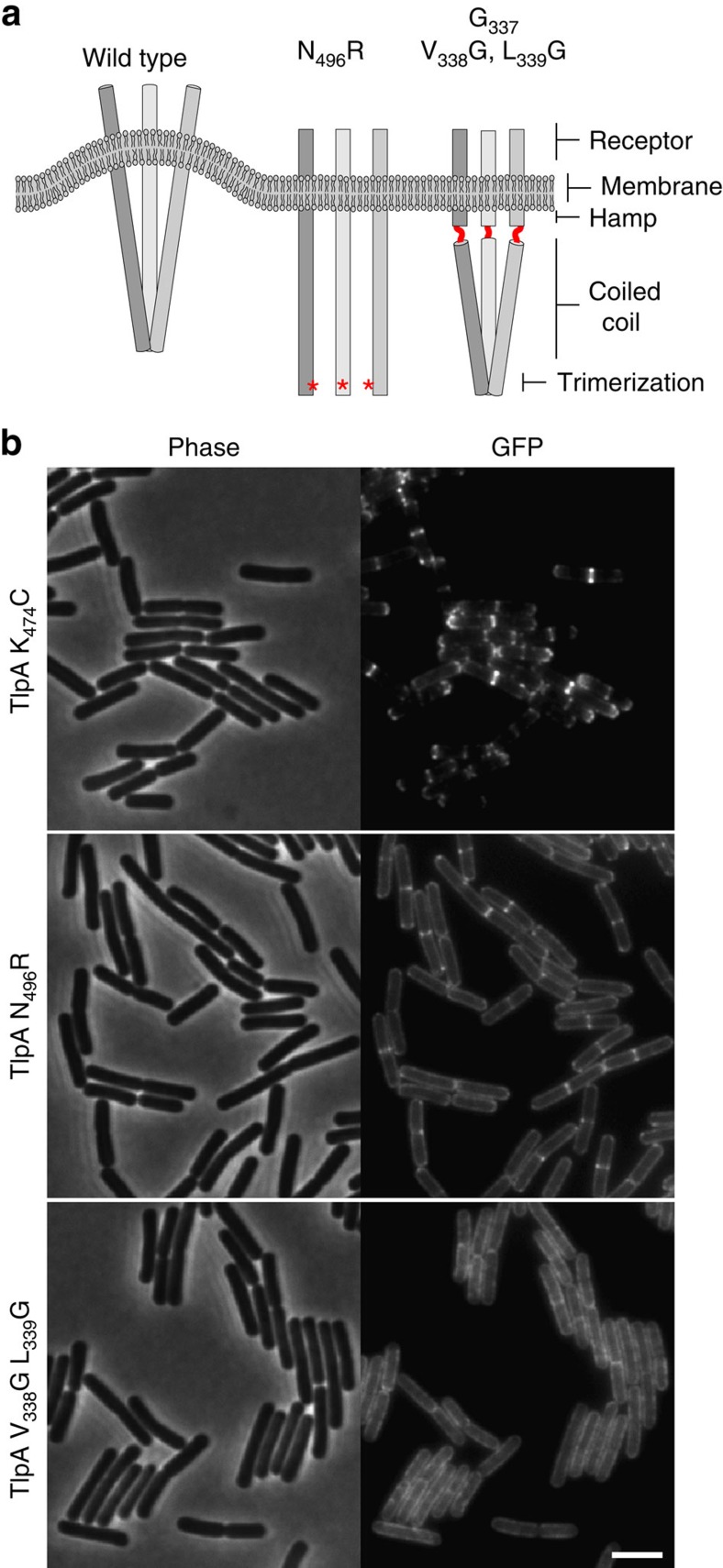
Localization of TlpA depends on the shape of the protein complex. (**a**) Schematic model of a TlpA trimer of dimers bending the cell membrane and the consequences of the different mutations (see main text for details). (**b**) Phase-contrast (left panel) and fluorescence (right panel) images of *B. subtilis* cells expressing TlpAK_474_C-GFP (cysteine substitution used for crosslinking), TlpAN_496_R-GFP (no trimerization) and TlpAV_338_G, L_339_G-GFP (increased flexibility). Strains used: *B. subtilis* HS58 (Δ*mcp Pxyl-tlpA(K*_*474*_*C)-gfp*), *B. subtilis* HS59 (Δ*mcp Pxyl-tlpA(K*_*474*_*C, N*_*496*_*R)-gfp*) and *B. subtilis* HS60 (Δ*mcp Pxyl-tlpA(K*_*474*_*C, V*_*338*_*G, L*_*339*_*G)-gfp*). Scale bar, 3 μm.

If the tripod-like shape of the chemoreceptor trimer of dimers, which causes a curved configuration of the complex, is indeed responsible for localization, then mutations that prevent trimerization should result in delocalized chemoreceptors (Figs 5a and 6a). To test this, we introduced the N_496_R substitution at the conserved trimerization interface of TlpA (Fig. 5a,b). This substitution has been shown to inhibit trimer formation of *E. coli* chemoreceptor Tsr dimers^33^. To confirm that this substitution inhibits trimerization of TlpA dimers, we introduced a cysteine residue at position 474 to enable cysteine crosslinking, which was previously used to show trimerization of chemoreceptors^33^,^34^. Importantly, this cysteine exchange does not alter the cellular localization of TlpA (Fig. 6b). Subsequent *in vivo* crosslinking using the tri-functional maleimide crosslinker tris(2-maleimidoethyl) amine established that the N_496_R exchange indeed inhibits trimerization of TlpA dimers (Fig. 5c). When the mutant protein was introduced into *B. subtilis*, the fluorescent signal became diffuse over the membrane and there was no significant enrichment at cell division sites (Fig. 6b).

As an additional test, we constructed a stretch of three glycines at the interface (position 337–339) between the coiled-coil domain and the HAMP domain of TlpA. Introduction of this glycine stretch provides high torsional flexibility in the dimers^35^,^36^, which should remove the preference for curved membranes (Fig. 6a). Cysteine crosslinking and western blot analysis showed that the introduction of the glycines does not interfere with the formation of trimer of dimers (Fig. 5c). However, as shown in Fig. 6b, the flexibility introduced into the dimers completely abolished normal localization. In conclusion, the septal accumulation of TlpA requires the formation of a rigid dimer-of-trimer complex. These observations strongly support a model whereby localization is based on the intrinsic curved shape of the protein complex.

## Discussion

On the basis of extensive microscopic studies with *E. coli*, Thiem *et al.*^12^,^14^ formulated a model for polar chemoreceptor clustering that relies on stochasticity driven formation of clusters. They assumed that the regular spacing between clusters is established by tethering to a so far unknown pre-divisional structure. Later studies by Greenfield *et al.*^13^ and Wang *et al.*^15^ showed that the periodicity of protein clusters can arise spontaneously from this stochastic nucleation mechanism. In an alternative model postulated by Endres^16^, the curved shape of large chemoreceptor clusters provides the force that drives the chemoreceptors to the (curved) cell poles. Our data indicate that neither of these models is sufficient to explain the polar localization of the classical chemoreceptor protein TlpA in *B. subtilis*. Although we do observe chemoreceptor clusters at the lateral cell wall, which are presumably formed by a form of stochastic nucleation, they are too frequent (per cell length), they are not periodically spaced and they do not show any accumulation/clustering at potential cell division sites. We have also shown that TlpA clusters by themselves cannot directly accumulate at cell poles as the ‘curved cluster’ model predicts^16^. However, from our data, it is apparent that the curved shape of the chemoreceptors is crucial for TlpA localization. The curvature at cell poles does not differ greatly from the curvature of the lateral cell wall. However, cell division creates regions with a distinct and strong curvature. This is for chemoreceptor trimer of dimers an energetically favourable environment due to a reduced membrane stress, and leads to the accumulation of chemoreceptors at cell division sites. The resulting high local concentration of trimer of dimers promotes the formation of large sensory arrays stabilized by CheA and CheW. Due to their large size, the lateral diffusion of these arrays is reduced and they remain localized at the new cell poles on completion of cell division, thus resulting in the canonical polar localization pattern. Possibly, the curved cluster mechanism described by Endres^16^ further contributes to this stable polar localization.

The different localization mechanisms in *E. coli* and *B. subtilis* might be related to the way these bacteria divide. In most Gram-positive bacteria including *B. subtilis*, cell division leads to the formation of a cross-wall, thereby creating a strongly curved cell membrane at midcell. On the other hand, *E. coli* cells constrict during division, resulting in a more moderate curvature of the cell membrane. A membrane curvature sensing mechanism seems therefore more adequate in species that generate local areas in the cell membrane with strong curvature.

Most bacteria contain chemoreceptors, and considering their vast variety and long evolutionary history, it is not surprising to find species that use other localization mechanisms. An interesting case is the localization of chemoreceptors in *Rhodobacter sphaeroides*. This organism undergoes large changes in the overall cell morphology, and possess a very atypical membrane topology with extensive invaginations of the membranes, which are related to its phototrophic lifestyle^37^. *R. spheroides* encodes a highly complex chemosensory system with both cytoplasmic as well as membrane chemoreceptor clusters^38^. In contrast to *E. coli* and *B. subtilis*, the membrane-associated chemoreceptors of *R. spheroides* form polar clusters after cell division is completed^39^. Possibly, the absence of chemoreceptors at the site of cytokinesis is related to the constriction-like division process of this ovoid bacterium, which might generate insufficient membrane curvature at the cell division site. Interestingly, *R. sphaeroides* cell poles are not perfectly round and contain a slightly protruding division scar^37^, thus creating a small area at the cell poles where chemoreceptors could accumulate in a curvature-dependent manner. Although speculative, it is conceivable that the remarkable changes in cell and membrane morphology could necessitate the use of an alternative mechanism in this organism. A novel mechanism of polar chemoreceptor targeting was discovered for the cytoplasmic chemoreceptor clusters in *R. sphaeroides*, and for both the cytoplasmic and membrane chemoreceptor clusters in *Vibrio cholera.* In these cases, the chemoreceptor clusters are segregated to cell poles by an active partitioning (Par) system, homologous to the Par systems involved in chromosome and plasmid segregation^40^,^41^,^42^. However, this mechanism is likely to have a relatively narrow taxonomic distribution^40^,^41^.

A remaining question is whether the cell benefits from polarly localized chemoreceptors. Several studies have shown that clustering of chemoreceptors increases the overall sensitivity for attractants/repellents by amplifying the chemotactic stimuli through extensive allosteric interactions among receptors^8^,^9^. The preference for highly curved septal membranes creates a high local concentration of chemoreceptors that promotes the formation of these sensory clusters. Because of the bilateral symmetry of curved membrane areas at division sites, the curvature-driven localization mechanism also ensures that both daughter cells inherit a chemoreceptor cluster. Taken together, it is conceivable that polar localization pattern is simply a consequence of the mechanism used to stimulate cluster formation by local enrichment, and to ensure stable inheritance to daughter cells.

**Figure 7:**
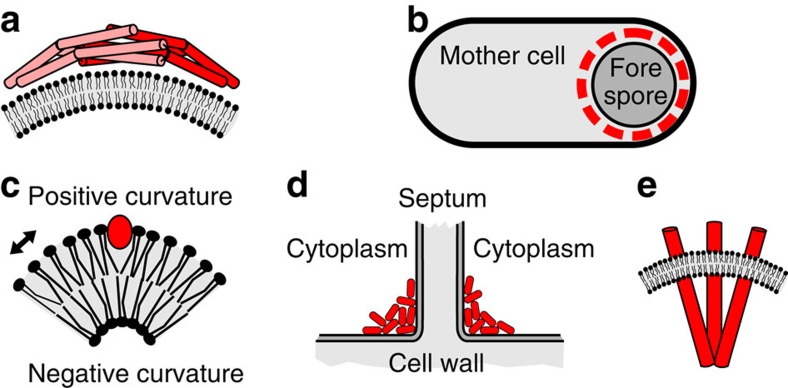
Different mechanisms to localize at curved membranes. (**a**) Schematic depiction of the BAR domain containing Arfaptin dimer using the ‘scaffolding’ mechanism to generate and sense membrane curvature. (**b**) Sporulating *B. subtilis* cell with SpoVM (red) coated forespore. (**c**) Insertion of an amphipathic helix (red) at the positively curved lipid bilayer face. Arrow indicates increased spacing. (**d**) Accumulation of DivIVA (red) at negatively curved membranes at the base of a cell dividing septum. (**e**) Membrane curvature generated by the chemoreceptor trimer-of-dimers shape.

Several membrane proteins can use local curvature of the membrane as a cue for localization. However, this mechanism has so far only been described for peripheral membrane proteins. Prominent examples are eukaryotic Bar domain proteins and proteins carrying an ALPS motif^43^, and the bacterial proteins DivIVA and SpoVM^24^,^25^,^44^,^45^. Bar domain containing proteins (for example, endophilins and amphiphysins) are involved in endocytosis and other membrane remodelling processes. The classic BAR domain structure is a curved dimer with a characteristic ‘banana shape’ that binds and bends lipid bilayers (Fig. 7a). Most of these proteins have a high affinity for positively curved membranes based on their shape (scaffold mechanism) and/or the insertion of amphipathic helices into the bilayer^43^,^46^. The latter mechanism is analogous to the binding of the amphipathic SpoVM helix. During sporulation, the developing spore (forespore) matures inside the *B. subtilis* mother cell (Fig. 7b). The spore coat protein SpoVM is able to distinguish these topological distinct membrane areas and only covers the membrane surface of the forespore (Fig. 7b)^47^. SpoVM is structurally an amphipathic helix and, like other amphipathic helices such as the ALPS motif-containing proteins^43^, has a preference for positively curved membranes due to reduced lipid-packing density in the outer leaflet that facilitates accommodation of a bulky α-helix^48^ (Fig. 7c). A different case is the protein DivIVA that functions as a scaffold for proteins involved in cell division in Gram-positive bacteria. The protein has a strong preference for negatively curved membrane areas at the base of the developing division septum, much like TlpA does^24^,^25^ (Fig. 7d). DivIVA does not have a specific curved shape but Monte Carlo simulations showed that weak interactions between the large DivIVA multimers, and between DivIVA and the cell membrane, are sufficient to cause accumulation at strong negatively curved membranes^24^. This so called ‘molecular bridging’ mechanism requires 3D (cytoplasmic) diffusion and can therefore not account for the localization of transmembrane proteins.

Several *in silico* studies have suggested that transmembrane proteins can accumulate at curved membrane areas in case their intrinsic shape distorts the lipid bilayer sufficiently^49^,^50^,^51^. The mitochondrial dimeric F_1_F_o_ ATP synthase is found enriched at the highly curved mitochondrial membrane, and a shape mismatch between protein and membrane plane is put forward as a potential explanation for this localization pattern^52^. A recent *in vitro* study using an artificial curved membrane system and a membrane distorting Kv voltage-gated ion channel protein provided further support for this hypothesis^30^. Here we show that this protein localization mechanism is indeed active *in vivo*. Since distinct curved membrane areas are ubiquitous, it will be interesting to see how common this morphogenetic process is.

## Methods

### Bacterial strains and growth conditions

Strains and conditions for gene induction are listed in Supplementary Table 1. *B. subtilis* was grown in LB medium at 30 °C. *B. subtilis* L-forms were grown in nutrient broth supplemented with sucrose, Mg^2+^, maleic acid and PenG as described earlier^31^. All experiments were performed with early-mid logarithmic growth phase cultures.

### Construction of strains

For the construction of chromosomal TlpA-mGFP fusion, the integration plasmid pSG1154-encoding eGFP was converted into a variant encoding monomeric GFP(A_206_K) to prevent dimerization of GFP^17^. The *in vivo* functionality of this fusion cannot be verified because the attractants or repellents of this chemoreceptor are unknown. The mutagenesis was performed by quick change method using primers GFP(A_206_K)-for/GFP(A_206_K)-rev resulting in plasmid pHJS102. The TlpA-mGFP fusion was constructed by PCR amplification of *tlpA* from *B. subtilis* chromosomal DNA using primers TlpA-for/TlpA-rev, linearization of plasmid pHJS102 using primers pSG1154-for/pSG1154-rev, and by subsequent fusion of the PCR products using In-Fusion HD Plus restriction free cloning kit (Clontech). The resulting plasmid pHJS102 TlpA-mGFP was further mutagenized with quick change method to introduce the point mutations K_474_C, N_496_R and V_338_G, L_339_G with primers listed in Supplementary Table 2. For the construction of chromosomal mCherry-CheA fusion, *mcherry* and *cheA* were amplified using primers mCherry-for/mCherry-rev and CheA-for/CheA-rev, followed by restriction digests with SalI/BamHI and BamHI/EcoRI, respectively, and ligation into pAPNC-kan. The plasmids were sequenced, and integrated into the *amyE* or *aprE*-loci of the recipient strains as described earlier^53^.

### Wide-field microscopy

Wide-field fluorescence microscopy was performed with cells immobilized on microscope slides covered with a thin film of 1.2% agarose. Unless stated differently, bacterial cell membranes were visualized with Nile Red at a final concentration of 1 μg ml^−1^. Microscopy was carried out with Zeiss Axiovert 200M (Zeiss Plan-Neofluar × 100/1.30 Oil Ph3 objective, Photometrics CoolSnap HQ2 CCD camera), Nikon Eclipse Ti (Nikon Plan Fluor × 100/1.30 oil Ph3 DLL objective, Rolera EM-C2 EMCCD camera) and Applied Precision DeltaVision RT (Zeiss Plan-Neofluar × 100 Ph3 objective, Photometrics CoolSnap HQ CCD camera). The images were acquired with Metamorph 6 (Molecular Devices) and softWoRx Suite (Applied Precision), and further analysed using ImageJ 1.48 (NIH). Deconvolution was carried from optical sections using Huygens Essentials v.3.3 (Scientific Volume Imaging).

### Confocal microscopy

Confocal microscopy was carried out with Nikon Ti equipped with a spinning-disk confocal module (Yokogawa CSU22), 491 nm/50 mW solid-state laser (Cobolt Dual Calypso), Nikon Plan Apo × 100/1.40 oil objective, and Rolera EM-C2 EMCCD camera. The images were acquired with Frap-AI 7.7.5.0 (MAG Biosystems) and analysed using ImageJ 1.48 (NIH). Cell sample preparation was as described for wide-field microscopy.

### Structured illumination microscopy

Dual-colour SIM was performed using Nikon N-SIM equipped with Nikon CFI APO TIRF × 100/1.49 oil objective, 488 nm (Coherent Sapphire) and 561 nm (Cobolt Jive 100) solid-state lasers, and Andor Xion X3 EMCCD camera. Image capture and reconstruction of high-resolution 3D SIM images was performed with NIS elements 4.0 (Nikon). The cells were immobilized on 1.2% agarose slides as described above. To reduce the binding of hydrophobic membrane dyes on the coverslip surface, which interferes with the projection of structured illumination pattern, the coverslips were coated with L-dopamine^54^. The coating was performed by addition of a large drop of freshly solved L-dopamine (2 mg ml^−1^ in 1 mM Tris pH 8.0) to a coverslip surface followed by 30-min incubation at room temperature. Subsequently, the non-polymerized L-dopamine and Tris were removed by aspiration and submersion of the coverslip in H_2_O, followed by evaporation at 37 °C for 30 min.

### *In vivo* crosslinking

For the *in vivo* crosslinking, cultures expressing different variants of TlpA-GFP were cultivated to an OD_600_ of 0.5, rapidly chilled on wet ice, washed with and resuspended in ice cold PBS, and subsequently flash-frozen in liquid N_2_. The cell suspensions were crosslinked with a tri-functional maleimide crosslinker tris(2-maleimidoethyl) amine (Pierce) at a concentration of 2 mg ml^−1^ (10-min incubation at 30 °C under shaking). The crosslinking was subsequently blocked by the addition of 20 mM cysteine. Samples for SDS–polyacrylamide gel electrophoresis and western blotting were prepared with 10-min incubation with lysozyme at 30 °C, followed by brief sonication and dilution in SDS gel-loading buffer. SDS–polyacrylamide gel electrophoresis was performed with Novex NuPAGE 4–12% Bis-Tris Midi Gels (Life Technologies), western Blotting with polyvinylidene difluoride membrane (GE Healthcare), and detection of bound antibodies with ECL 2 Western Blotting Substrate (Pierce). The used antibodies were polyclonal rabbit α-GFP_6His_ (1:3,000, laboratory stock) and monoclonal goat α-rabbit HRP (1:10,000, Sigma).

### Structure modelling

The structural modelling of TlpA was performed with Phyre 2 (ref. 55). No structure of a full-length chemoreceptor has been resolved so far. Therefore, TlpA amino acids 44–283 (receptor domain) were modelled using structure 3C8C (Phyre2 confidence estimate 99%) as a template (MCP from *Vibrio cholera*, Patskovsky *et al.* unpublished). Amino acids 304–661 (coiled coil+HAMP domain) were modelled using the *E. coli* Tsr structure^56^ (Phyre2 confidence estimate 99%). The overall trimer-of-dimer structure is based on the corresponding structure of *E. coli* Tsr^57^. The amino acids 1–44 and 284–303 represent two transmembrane domains of the protein. These domains were modelled as ideal α-helices connecting the extracellular receptor domains and the cytoplasmic domains using DeepView/Swiss-PdbViewer^58^. The second transmembrane domain is modelled as an α-helix, which is continuous with an α-helix from the HAMP domain, but discontinuous with an α-helix from the receptor domain. The orientation of the receptor domain with respect to the rest of the protein is therefore speculative, but compatible with tomographic images of chemoreceptor complexes^59^.

## Additional information

**How to cite this article:** Strahl, H. *et al.* Transmembrane protein sorting driven by membrane curvature. *Nat. Commun.* 6:8728 doi: 10.1038/ncomms9728 (2015).

## Supplementary Material

Supplementary Information Supplementary Figures 1-11, Supplementary Tables 1-2 and Supplementary References
