# Supplementary material for: Transmembrane protein sorting driven by membrane curvature
Source: Nat Commun. 2015 Nov 2;6:8728. doi: 10.1038/ncomms9728 (PMC4632190; doi:10.1038/ncomms9728)
Supplement: Supplementary Information — Supplementary Figures 1-11, Supplementary Tables 1-2 and Supplementary References [file ncomms9728-s1.pdf]

SUPPLEMENTARY FIGURES

Supplementary Figure 1

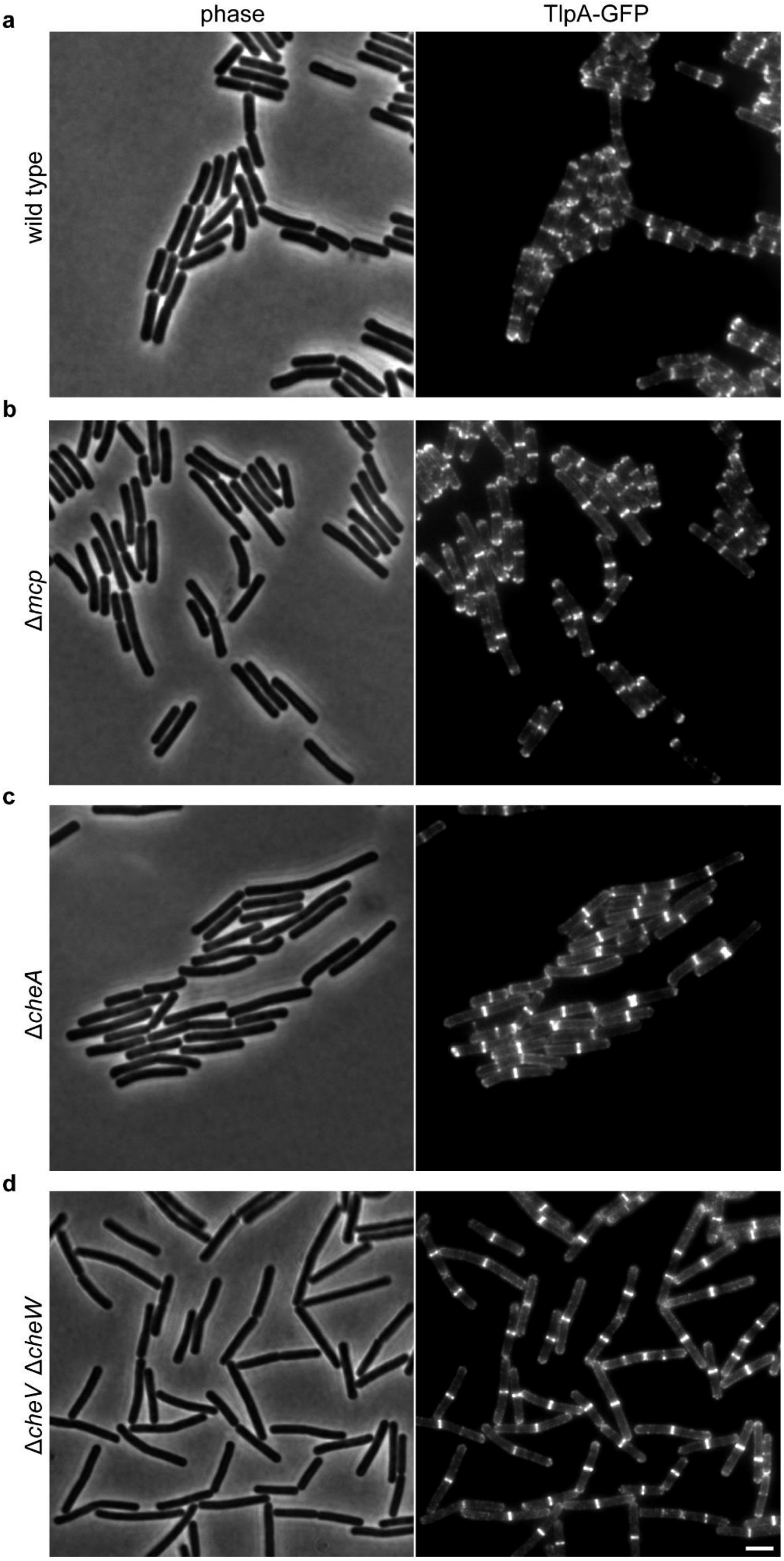

***B. subtilis* TlpA localizes at cell poles and cell division sites**

Phase contrast images (left panels) of *B. subtilis* cells expressing TlpA-GFP (right panels) in the presence (a) and absence (b) of other MCPs, and in the absence of CheA (c) or CheV and CheW (d). No difference in recruitment to the cell division sites is observed in the different strain backgrounds. In contrast, the polar localization is reduced in the absence of CheA, or CheV and CheW. Note that *B. subtilis* encodes two CheW-homologs (CheW and CheV). These are larger fields of cells shown in Fig. 1 and Fig. 2d. Strains used: *B. subtilis* HS48 (*Pxyl-tlpA-gfp*), *B. subtilis* HS49 (Δ*mcp Pxyl-tlpA-gfp*), *B. subtilis* HS53 (Δ*cheA Pxyl-tlpA-gfp*), and *B. subtilis* HS63 (Δ*cheV* Δ*cheW Pxyl-tlpA-gfp).* Scale bar, 3 μm.

Supplementary Figure 2

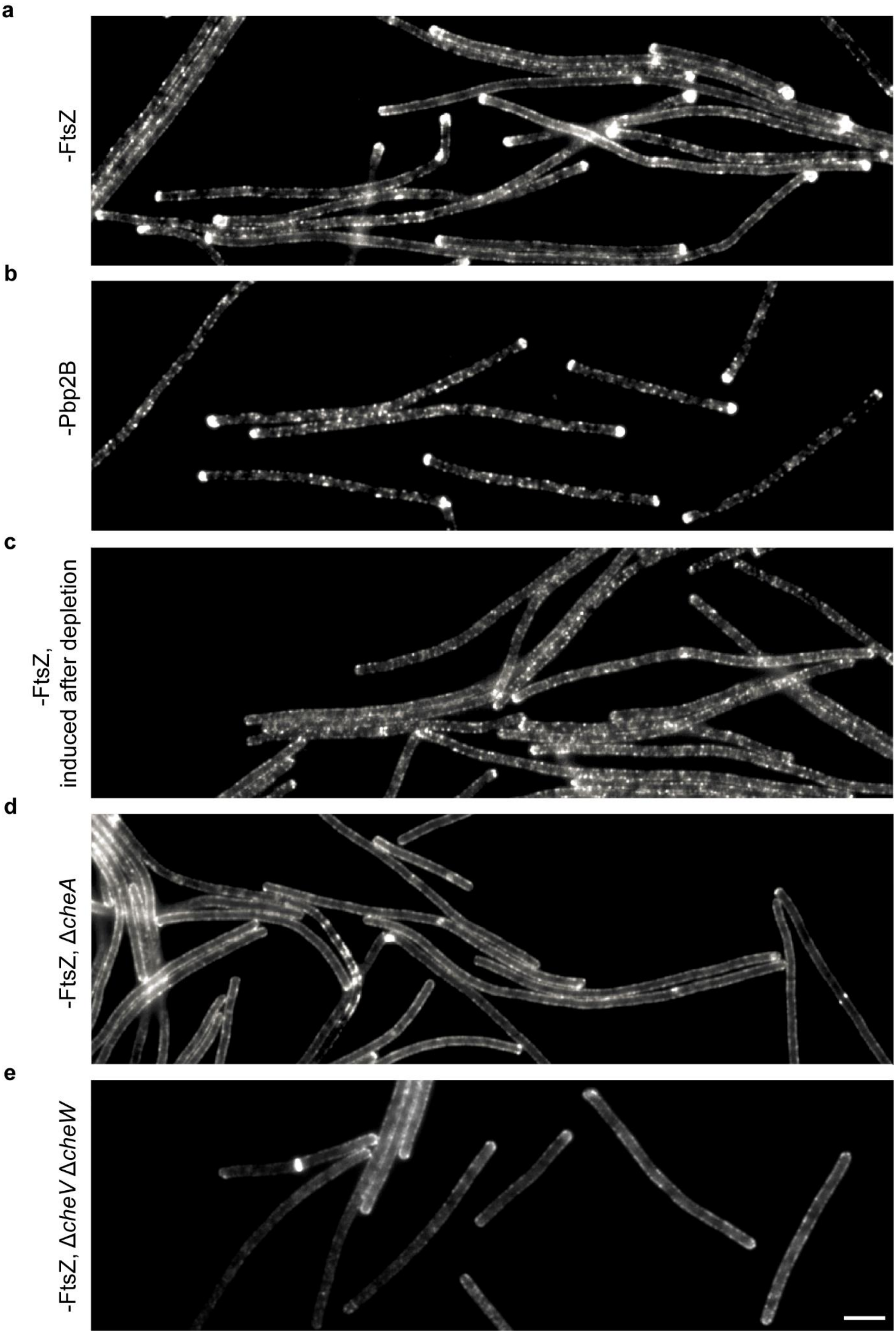

TlpA localizes to cell division sites

(a) Fluorescence images of *B. subtilis* expressing TlpA-GFP in cells depleted for FtsZ, and (b) in cells depleted for Pbp2B in the absence of other MCPs. (c) Localization of TlpA-GFP in cells in which FtsZ was depleted *before* expression of *tlpA-gfp* was induced. (d) Localization of TlpA-GFP in cells depleted for FtsZ in the absence of CheA, and (e) in the absence of CheV and CheW. These are larger fields of cells shown in Fig. 2a, 2b, and 2c. Strains used: *B. subtilis* HS50 (Δ*mcp Pxyl-tlpA-gfp Pspac-ftsZ*), *B. subtilis* HS51 (Δ*mcp Pxyl-tlpA-gfp Pspac-pbpB*), *B. subtilis* HS54 (Δ*cheA Pxyl-tlpA-gfp, Pspac-ftsZ*), and *B. subtilis* HS64 (Δ*cheV* Δ*cheW Pxyl-tlpA-gfp, Pspac-ftsZ*). Scale bar, 3 µm.

Supplementary Figure 3

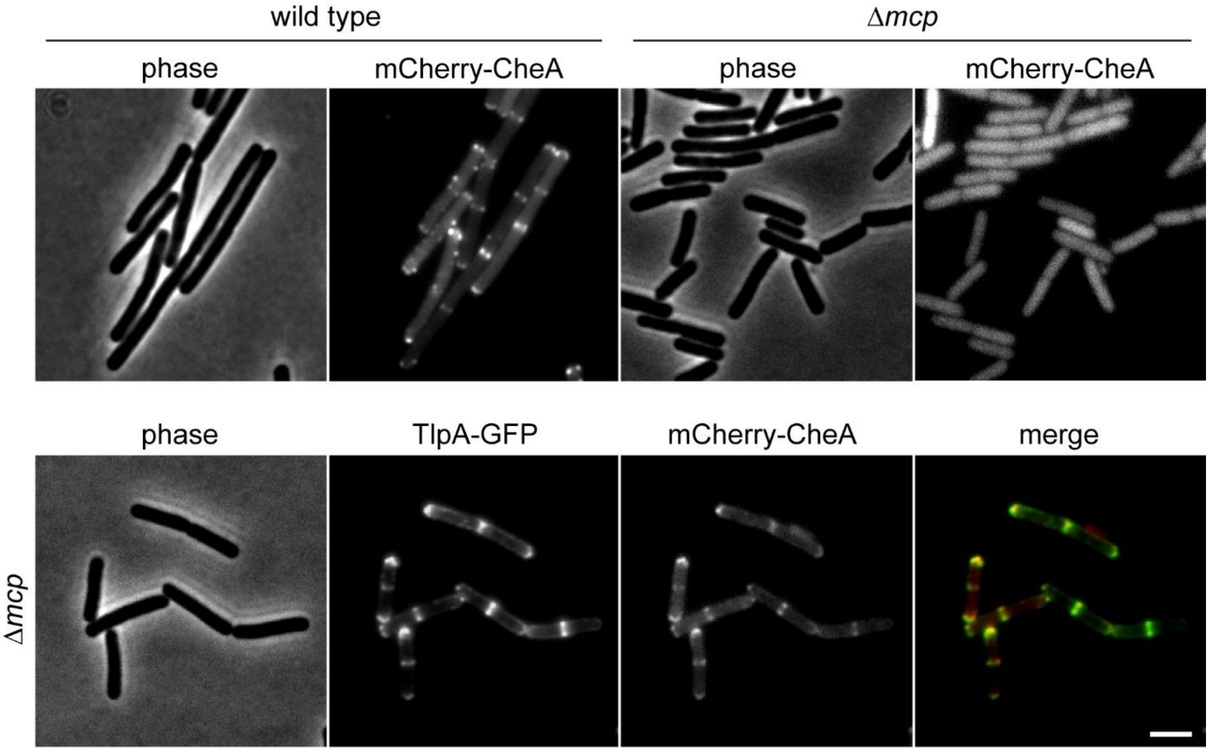

Cellular localization of CheA

Cellular localization of mCherry-CheA in wild type cells, in a strain deficient for all 10 native chemoreceptors (upper panels), and in the presence of TlpA-GFP (lower panels). Note that the localization in wild type cells is comparable to that of TlpA and that there is no localization in the absence of chemoreceptors. When only TlpA-GFP is present in the Δ*mcp* background strain, the localization of mCherry-CheA is restored (lower panel). Strains used: *B. subtilis* HS65 (*Pspac-mcherry-cheA*), *B. subtilis* HS66 (Δ*mcp Pspac-mcherry-cheA*), and *B. subtilis* HS67 (Δ*mcp Pspac-mcherry-cheA Pxyl-tlpA-gfp*). Scale bar, 3 μm.

Supplementary Figure 4

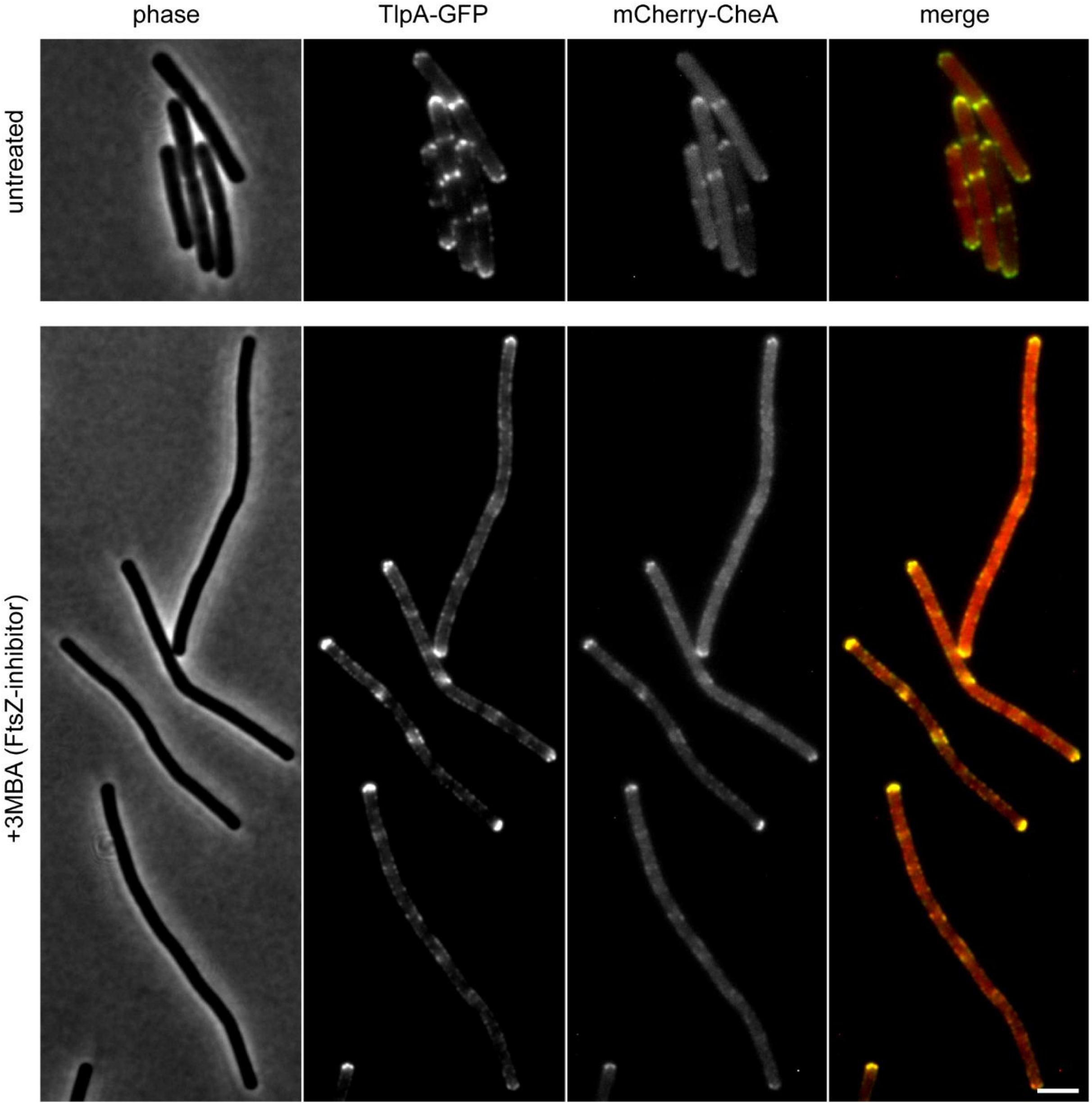

Colocalization of TlpA and CheA in the presence and absence of cell division

A strain co-expressing TlpA-GFP and mCherry-CheA is depicted in the absence (upper panels) and presence (lower panels) of the FtsZ-inhibitor 3MBA (3-Methoxybenzamide) [1]. There is a clear colocalization of TlpA with CheA both at the cell poles, and in the lateral membranes of the division-inhibited cells. Strain used: *B. subtilis* HS67 (Δ*mcp Pspac-mcherry-cheA Pxyl-tlpA-gfp*). Scale bar, 3 µm.

Supplementary Figure 5

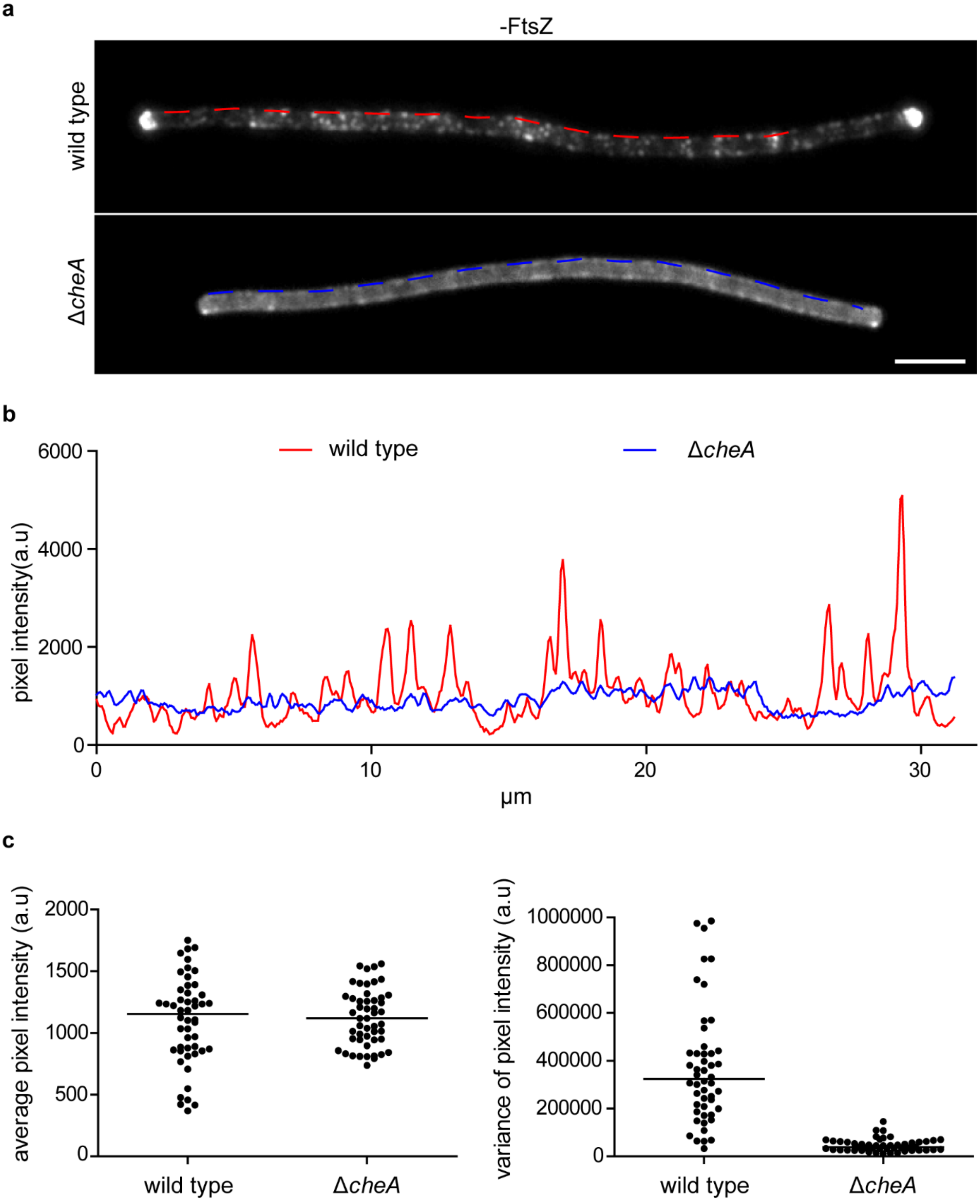

Chemoreceptor clusters in the absence of cell division are CheA-dependent

The clustering of TlpA in non-dividing cells in the presence and absence of CheA was quantified using an intensity line scan analysis. (a) Fluorescence images of representative FtsZ-depleted cells are shown in the presence and absence of CheA. (b) A fluorescence intensity line

scan along the membrane plane (dotted line show in panel A) is depicted. Note the clear clustering of the fluorescent signal (peaks) in the presence of CheA. (c) The average and variance of fluorescence intensity of a line scan measured for 50 individual cells. The unchanged average signal indicates comparable overall fluorescent protein levels in the membrane (left panel). The clear difference in pixel intensity variance indicates a significantly altered clustering (right panel) [2,3]. In conclusion, the observed foci are CheA-dependent clusters rather than individual chemoreceptor trimers. Strains used: *B. subtilis* HS68 (*Pxyl-tlpA-gfp, Pspac-ftsZ*), and *B. subtilis* HS54 (Δ*cheA Pxyl-tlpA-gfp*, *Pspac-ftsZ*). Scale bar, 3 μm.

Supplementary Figure 6

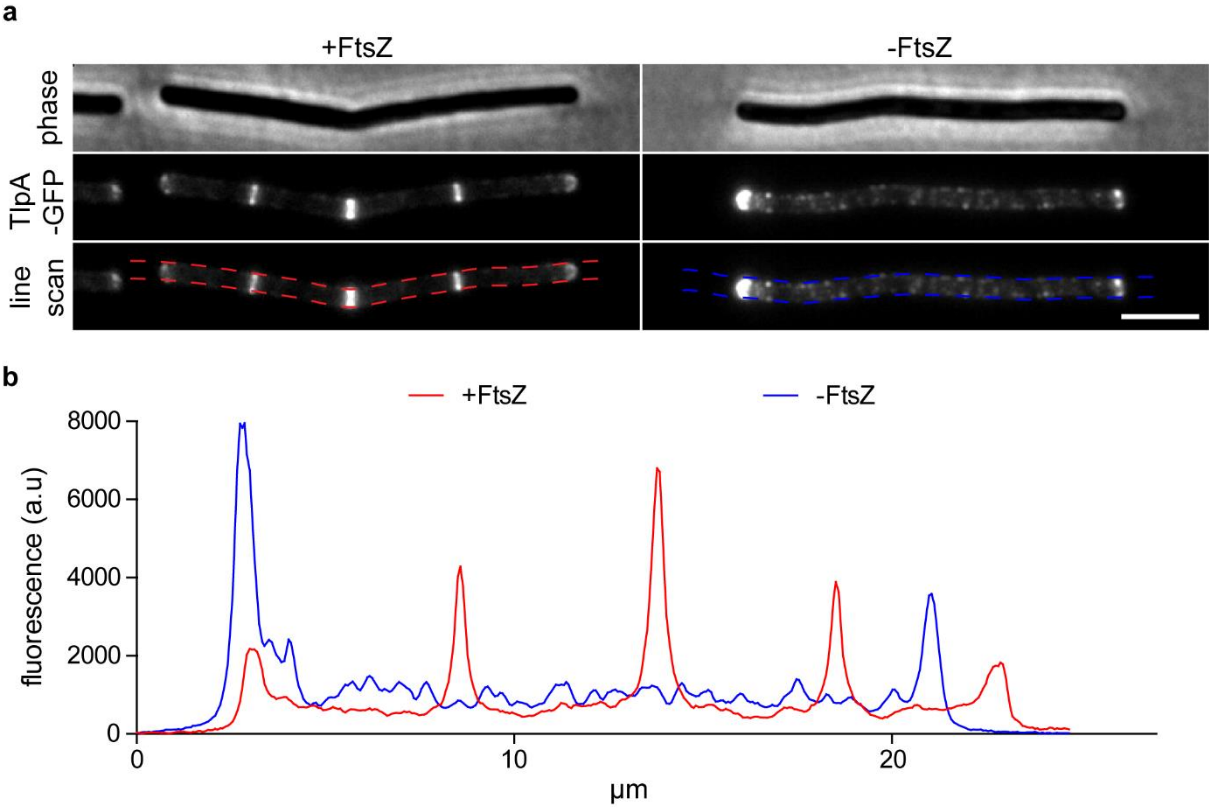

Chemoreceptor clustering follows a periodic pattern only in dividing cells

(a) The periodic nature of chemoreceptor clustering was analysed in dividing (+FtsZ) and non-dividing (-FtsZ) cells using a line scan analysis. (b) A longitudinal fluorescence intensity profile of representative cells depicted in panel a was generated by averaging 14 pixels (0.9 µm) aligned perpendicular to the cell length axis, and by measuring an intensity profile along the length axis of the cell. Note the periodic pattern of clustering in dividing cells which is absent in cells depleted for FtsZ. Strain used: *B. subtilis* HS50 (Δ*mcp Pxyl-tlpA-gfp Pspac-ftsZ*). Scale bar, 3 µm.

Supplementary Figure 7

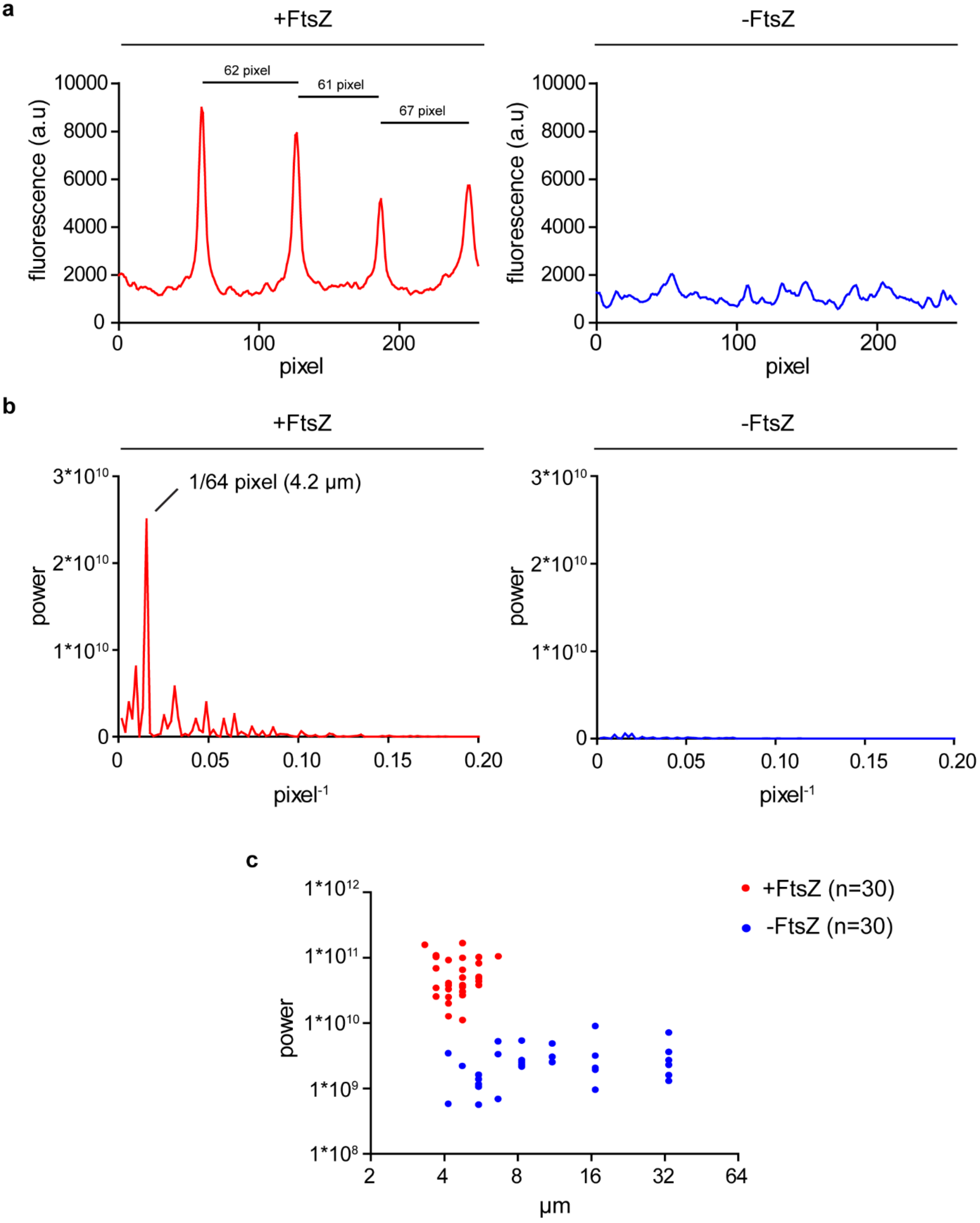

Fast Fourier transform analysis of periodic chemoreceptor clustering

Fast Fourier transform (FFT) analysis is a mathematical method for detecting periodicities in a signal. FFT can be applied to identify periodic localization patterns in microscopic data [4,5], and was performed here to analyse the periodicity of chemoreceptor clustering in the presence and absence of cell division. (a) Fluorescence intensity line scans of representative cells in the presence and absence of FtsZ. The measurement of intensity was essentially carried out as described in Fig. S4. In this case, the cell poles were omitted in order to specifically analyse the periodicity of non-polar clustering. (b) Fast Fourier transformed intensity profiles for the data shown in panel a are depicted as signal power (square of absolute amplitude) plotted against frequency (1 per pixel). Note the strong periodicity in dividing cells that correlates with an average cell length. In contrast, no clear periodicity is detected in non-dividing cells. (c) The analysis was repeated for 30 depleted and non-depleted cells. The power of strongest frequencies is plotted against the wavelength of the corresponding periodic pattern. Note the strong periodicities correlating with an average cell length in dividing cells. In contrast, the detected periodicities in non-dividing cells are weak, and vary strongly between individual cells. In conclusion, no significant periodic pattern is detected for chemoreceptor clustering in the absence of cell division. Strain used: *B. subtilis* HS50 (Δ*mcp Pxyl-tlpA-gfp Pspac-ftsZ*).

Supplementary Figure 8

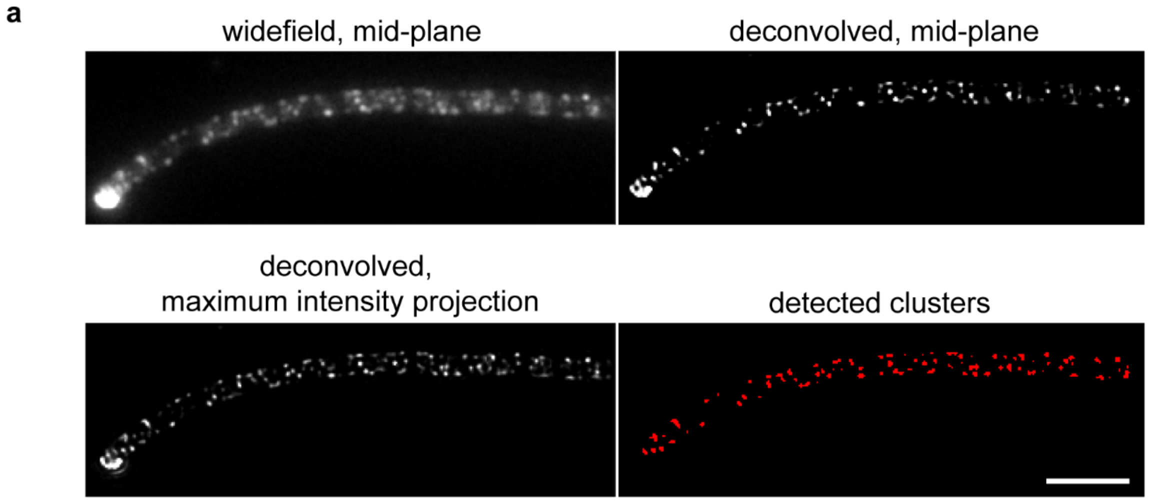

b

	clusters	cell length (µm)	clusters/µm	clusters/cell equivalent*
cell 1	231	50.3	4.6	23
cell 2	216	54.0	4.0	20
cell 3	267	53.3	5.0	25
cell 4	329	68.6	4.8	24
cell 5	140	35.8	3.9	19.5
cell 6	97	23.3	4.2	21
cell 7	254	49.4	5.1	25.5
cell 8	237	54.9	4.3	21.5
mean ± s.d.			**4.5** ± 0.5	**22.4** ± 2.3

*calculated with the average cell length of *B. subtilis* wild type under comparable growth conditions

Chemoreceptor clustering in the absence of cell division

The density of small chemoreceptor clusters upon FtsZ-depletion was quantified from optical sections. To identify individual clusters from different focal planes, the z-stacks were deconvolved followed by a maximum intensity projection. The individual clusters were detected and quantified from the maximum intensity projections using ImageJ. (a) A representative cell at different stages of the image analysis is depicted. (b) The mean cluster density is calculated as clusters per µm of cylindrical cell section, and per cell length equivalent of a dividing cell (5 µm). Strain used: *B. subtilis* HS50 (Δ*mcp Pxyl-tlpA-gfp Pspac-ftsZ*). Scale bar, 3 µm.

Supplementary Figure 9

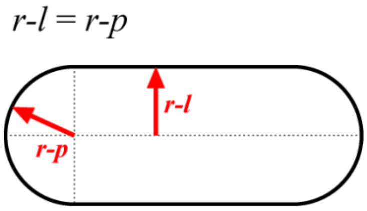

Schematic representation of the sphero-cylindrical geometry of a rod shaped bacterial cell

The radii of the cell pole (*r-p*) and the lateral part of the cell (*r-l*) are comparable and only differ in dimensionality (2-dimensional curvature at the cell pole, 1-dimensional curvature at the lateral part of the cell).

Supplementary Figure 10

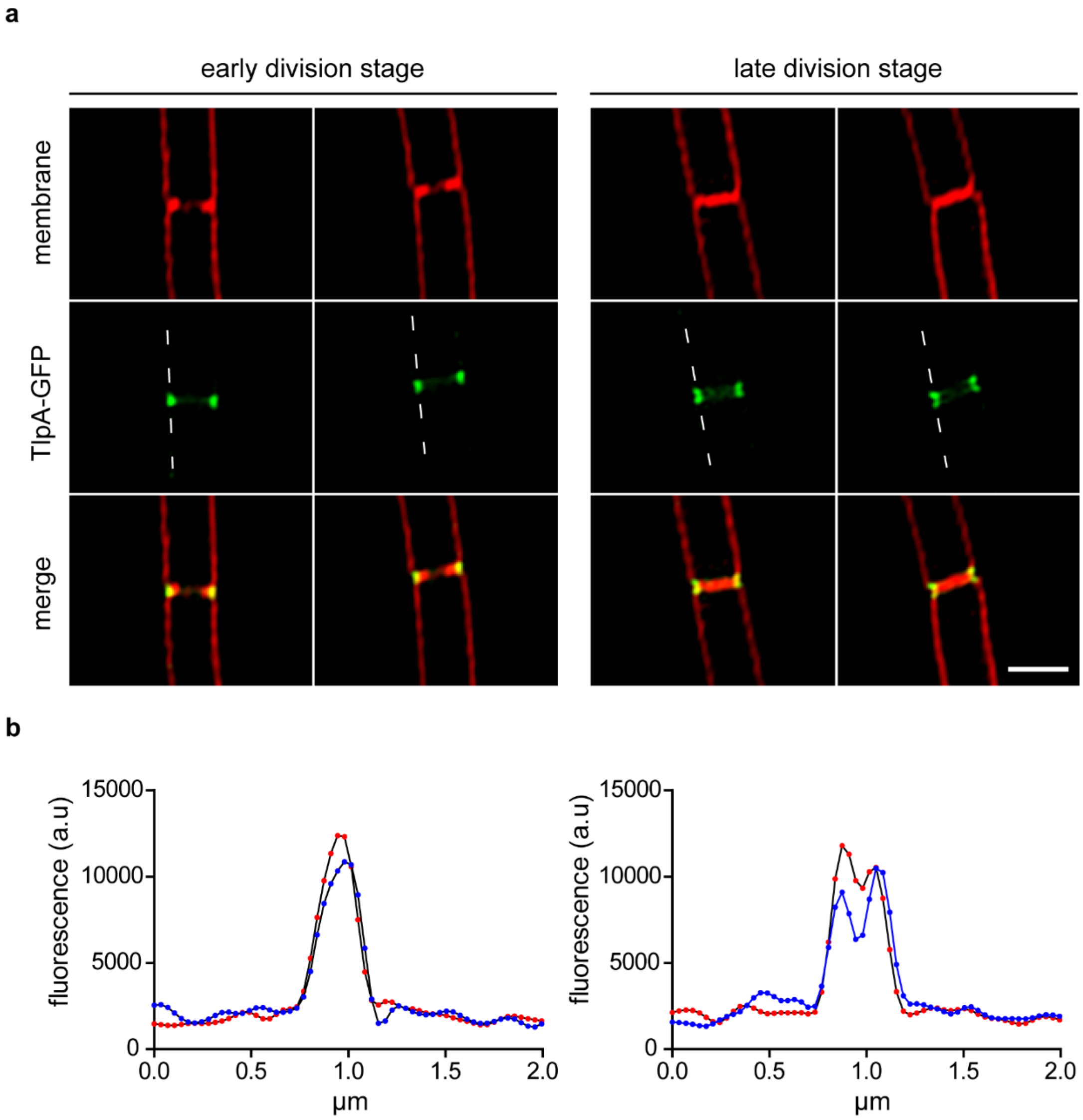

Septal localization of TlpA during early and later cell division stages

(a) Structured illumination microscopy (SIM) of TlpA-GFP and fluorescent membrane stain (Nile Red) is depicted in early, and late stages of cell division. Note the appearance of two septal TlpA-rings upon maturation of the cell division. (b) Fluorescent intensity line scans of the TlpG-GFP images depicted in panel a (the red and blue lines represent the two line scans for each condition shown in panel a). The point-spread function of TlpA in early dividing cells resolves into two individual peaks in later stage of the division. In conclusion, TlpA localizes symmetrically on both sides of the developing cell division plane (septum). Strain used: *B. subtilis* HS49 (Δ*mcp Pxyl-tlpA-gfp*). Scale bar, 1 µm.

Supplementary Figure 11

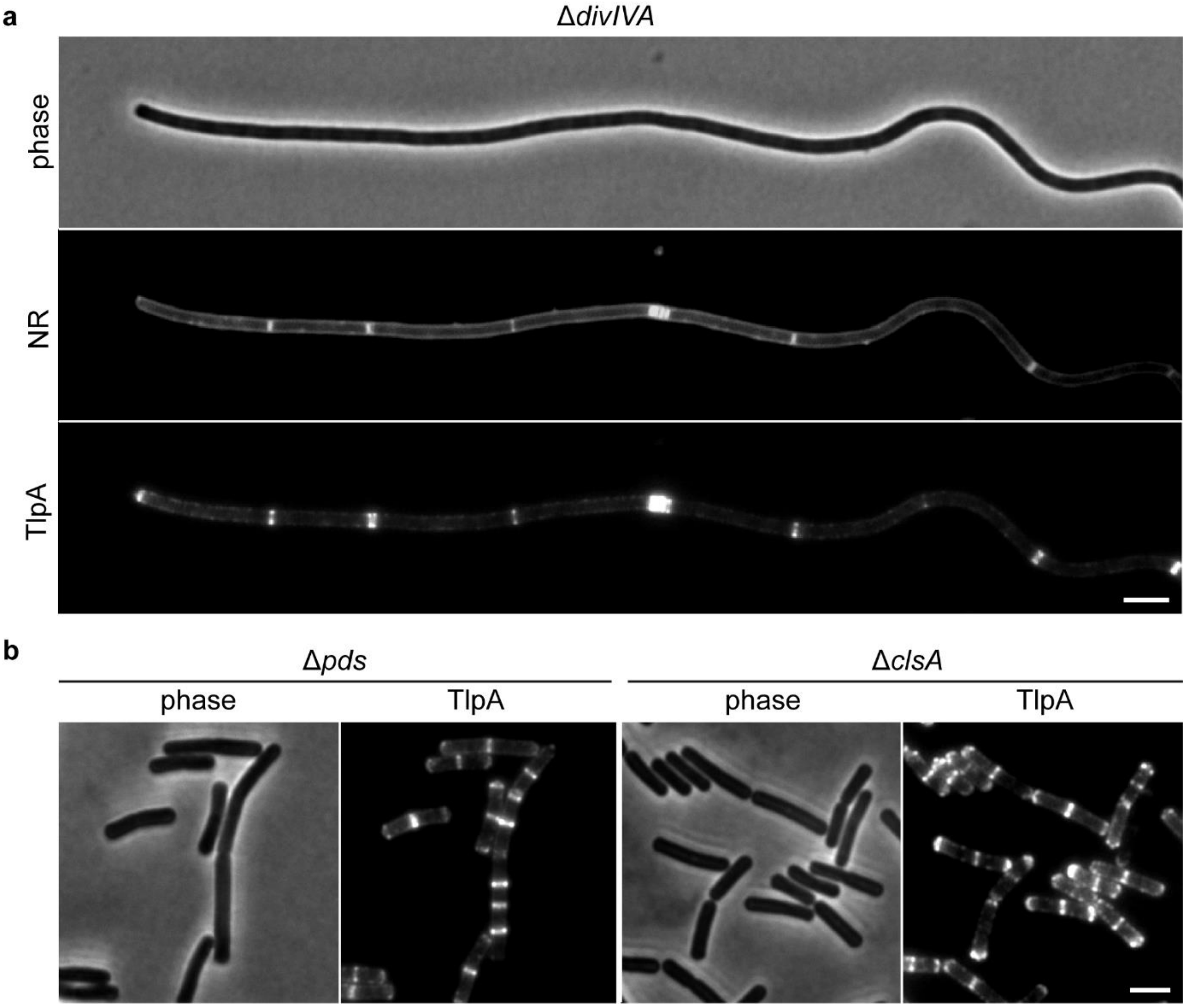

TlpA localization is independent of DivIVA, phosphatidylethanolamine and cardiolipin

(a) Phase contrast image of Δ*divIVA B. subtilis* cells (upper panel) expressing TlpA-GFP (lower panel), and fluorescent Nile Red membrane stains (middle panel). This is a wide field microscopy example of cells shown in Fig. 3c. (b) Phase contrast image of *B. subtilis* cells (left panels) expressing TlpA-GFP (right panels) in cells that do not synthesize phosphatidylethanolamine (Δ*pds*) or cardiolipin (Δ*clsA*). Strains used: *B. subtilis* HS52 (Δ*mcp* Δ*divIVA Pxyl-tlpA-gfp*), *B. subtilis* HS61 (Δ*pds Pxyl-tlpA-gfp*), and *B. subtilis* HS62 (Δ*clsA Pxyl-tlpA-gfp*). Scale bar, 3 µm.

Supplementary Table 1: Strains and Plasmids

Strain	Genotype/Properties	Induction		Source
B. subtilis 168	*trpC2* wild type	-		[6]
B. subtilis HS48	*amyE::spc pxyl-tlpa-mgfp*	0.3%	xyl	this work
B. subtilis OI3545	Δ10*mcp ery cat*	-		[7]
B. subtilis OI1840	*cheA::cat*	-		[8]
B. subtilis OI3061	*cheW::cat cheV::kan*	-		[9]
B. subtilis 1801	*Pspac-ftsZ ble*	0-100 µM	IPTG	[10]
B. subtilis 799	*ftsL::Pspac-pbpB kan*	0-1 mM	IPTG	[11]
B. subtilis HB5362	*clsA::cat*	-		[12]
B. subtilis HB5343	*pds::ery*	-		[12]
B. subtilis 4041	*divIVA::tet*	-		[13]
B. subtilis M96	*spoVD::cat Pxyl-murE*	0-1%	xyl	[14]
B. subtilis HS49	Δ10*mcp ery cat amyE::spc pxyl-tlpA-mgfp*	0.3 %	xyl	this work
B. subtilis HS50	Δ10*mcp ery cat amyE::spc pxyl-tlpA-mgfp*	0.3 %	xyl	this work
	Pspac-ftsZ ble	0-100 µM	IPTG	
B. subtilis HS51	Δ10*mcp ery cat amyE::spc pxyl-tlpA-mgfp*	0.3 %	xyl	this work
	ftsL::Pspac-pbpB kan	0-1 mM	IPTG	
B. subtilis HS52	Δ10*mcp ery cat amyE::spc pxyl-tlpA-mgfp*	0.3 %	xyl	this work
	divIVA::tet			
B. subtilis HS53	*cheA::cat amyE::spc pxyl-tlpA-mgfp*	0.3%	xyl	this work
B. subtilis HS54	*cheA::cat amyE::spc pxyl-tlpA-mgfp*	0.3%	xyl	this work
	Pspac-ftsZ ble	0-100 µM	IPTG	
B. subtilis HS55	*spoVD::cat Pxyl-murE sup*	0-1%	xyl	this work
	amyE::spc pxyl-tlpA-mgfp			
B. subtilis HS56	Δ10*mcp ery cat*	0.3%	xyl	this work
	amyE::spc pxyl-tlpA(N_{496}*R)-mgfp*			
B. subtilis HS57	Δ10*mcp ery cat*	0.3 %	xyl	this work
	amyE::spc pxyl-tlpA(V_{338}*G,* L_{339}*G)-mgfp*			
B. subtilis HS58	Δ10*mcp ery cat*	0.3%	xyl	this work
	amyE::spc pxyl-tlpA(K_{474}*C)-mgfp*			
B. subtilis HS59	Δ10*mcp ery cat*	0.3%	xyl	this work
	amyE::spc pxyl-tlpA(K_{474}*C,* N_{496}*R)-mgfp*			
B. subtilis HS60	Δ10*mcp ery cat*	0.3 %	xyl	this work
	amyE::spc pxyl-tlpA(K_{474}*C,* V_{338}*G,* L_{339}*G)-mgfp*			
B. subtilis HS61	*pds::ery amyE::spc pxyl-tlpA-mgfg*	0.3 %	xyl	this work
B. subtilis HS62	*clsA::cat amyE::spc pxyl-tlpA-mgfg*	0.3 %	xyl	this work
B. subtilis HS63	*cheW::cat cheV::kan amyE::spc pxyl-tlpA-mgfp*	0.3 %	xyl	this work
B. subtilis HS64	*cheW::cat cheV::kan amyE::spc pxyl-tlpA-mgfp*	0.3 %	xyl	this work
	Pspac-ftsZ ble			
B. subtilis HS65	*aprE::kan Pspac-mcherry-cheA*	1 mM	IPTG	this work
B. subtilis HS66	Δ10*mcp ery cat aprE::kan Pspac-mcherry-cheA*	1 mM	IPTG	this work
B. subtilis HS67	Δ10*mcp ery cat aprE::kan Pspac-mcherry-cheA*	0.3 %	xyl	this work
	amyE::spc pxyl-tlpA-mgfp	1 mM	IPTG	
B. subtilis HS68	*amyE::spc pxyl-tlpA-mgfp*	0.3 %	xyl	
	Pspac-ftsZ ble	0-100 µM	IPTG	

Plasmids	Genotype/Properties	Induction	Source
pSG1154	*bla amyE3' spc Pxyl gfpmut1 amyE5'*	-	[15]
pHJS102	*bla amyE3' spc Pxyl gfp(*A_{206}*K) amyE5'*	-	this work
pAPNC-kan	*bla aprE3' kan Pspac aprE5'*		De Olmedo Verd, unpubl.

Supplementary Table 2: Oligonucleotides

Oligonucleotide	Sequence
GFP(A_{206}K)-for	CCTGTCCACACAATCTAAACTTTCGAAAGATCCC
GFP(A_{206}K)-rev	GGGATCTTTCGAAAGTTTAGATTGTGTGGACAGG
TlpA-for	GAGATTCCTAGGATGAAAAAAACACTCACCACTATTC
TlpA-rev	TTCTCCTTTACTCATTTTGTCTACTTTAAATTGTTTTGTCAG
pSG1154-for	ATGAGTAAAGGAGAAGAACTTTTCAC
pSG1154-rev	CATCCTAGGAATCTCCTTTCTAG
TlpAK_{474}C-for	GTGAAAGGGCTGGAGATCAAATCATGCGATATCACGAATATTTTG
TlpAK_{474}C-rev	CAAAATATTCGTGATATCGCATGATTTGATCTCCAGCCCTTTCAC
TlpAN_{496}R-for	CCAATCTTTTGGCTTTACGTGCCGCCATTGAAGCTGCCAG
TlpAN_{496}R-rev	CTGGCAGCTTCAATGGCGGCACGTAAAGCCAAAAGATTGG
TlpAV_{338}G L_{339}G-for	GAACTCGGCGGTGGAAGTGAGAGCTTCAATCATATG
TlpAV_{338}G L_{339}G-rev	CATATGATTGAAGCTCTCACTTCCACCGCCGAGTTC
mCherry-for (*Sal*I)	GCGCGGTCGACACATAAGGAGGAACTACTATGGTC
mCherry-rev (*Bam*HI)	GCGCGGGATCCTGAGCCGCTTCCTGATTTGTATAATTCGTCCATTCCACC
CheA-for (*Bam*HI)	GCGCGGGATCCATGGATATGAATCAGTATTTAGATG
CheA-rev (*Eco*RI)	GCGCGGAATTCTTAAATAATCAGTGCATTACAATCAATAATG

SUPPLEMENTARY REFERENCES

1 Stokes, N. R. *et al.* An improved small-molecule inhibitor of FtsZ with superior *in vitro* potency, drug-like properties, and *in vivo* efficacy. *Antimicrob. Agents Chemother.* **57**, 317-325 (2013).

2 Strahl, H., Bürmann, F. & Hamoen, L. W. The actin homologue MreB organizes the bacterial cell membrane. *Nat. Commun.* **5**, 3442 (2014).

3 Strahl, H. *et al.* Membrane recognition and dynamics of the RNA degradosome. *PLoS Genet.* **11**, e1004961 (2015).

4 Cameron, T. A., Roper, M. & Zambryski, P. C. Quantitative image analysis and modeling indicate the *Agrobacterium tumefaciens* type IV secretion system is organized in a periodic pattern of foci. *PLoS ONE* **7**, e42219 (2012).

5 Zhong, G. *et al.* Developmental mechanism of the periodic membrane skeleton in axons. *Elife* **3**, doi: 10.7554/eLife.04581 (2014).

6 Barbe, V. *et al.* From a consortium sequence to a unified sequence: The *Bacillus subtilis* 168 reference genome a decade later. *Microbiology* **155**, 1758-1775 (2009).

7 Hou, S. *et al.* Myoglobin-like aerotaxis transducers in archaea and bacteria. *Nature* **403**, 540-544 (2000).

8 Fuhrer, D. K. & Ordal, G. W. *Bacillus subtilis* CheN, a homolog of CheA, the central regulator of chemotaxis in *Escherichia coli*. *J. Bacteriol.* **173**, 7443-7448 (1991).

9 Karatan, E., Saulmon, M. M., Bunn, M. W. & Ordal, G. W. Phosphorylation of the response regulator CheV is required for adaptation to attractants during *Bacillus subtilis* chemotaxis. *J. Biol. Chem.* **276**, 43618-43626 (2001).

10 Beall, B. & Lutkenhaus, J. FtsZ in *Bacillus subtilis* is required for vegetative septation and for asymmetric septation during sporulation. *Genes Dev.* **5**, 447-455 (1991).

11 Daniel, R. A., Williams, A. M. & Errington, J. A complex four-gene operon containing essential cell division gene *pbpB* in *Bacillus subtilis*. *J. Bacteriol.* **178**, 2343-2350 (1996).

12 Salzberg, L. I. & Helmann, J. D. Phenotypic and transcriptomic characterization of *Bacillus subtilis* mutants with grossly altered membrane composition. *J. Bacteriol.* **190**, 7797-7807 (2008).

13 van Baarle, S. *et al.* Protein-protein interaction domains of *Bacillus subtilis* DivIVA. *J. Bacteriol*. **195**, 1012-1021 (2013).

14 Leaver, M., Dominguez-Cuevas, P., Coxhead, J. M., Daniel, R. A. & Errington, J. Life without a wall or division machine in *Bacillus subtilis*. *Nature* **460**, 538-538 (2009).

15 Lewis, P. J. & Marston, A. L. GFP vectors for controlled expression and dual labelling of protein fusions in *Bacillus subtilis*. *Gene* **227**, 101-110 (1999).
